# Overage labor, intergenerational financial support, and depression among older rural residents: evidence from China

**DOI:** 10.3389/fpubh.2023.1219703

**Published:** 2023-08-23

**Authors:** Yongjie Chen, Kun Wang, Jinxu Zhao, Zixian Zhang, Jiangyin Wang, Li He

**Affiliations:** ^1^School of Public Administration, Hangzhou Normal University, Hangzhou, Zhejiang, China; ^2^Zhejiang Centre for Urban Governance Research, Hangzhou, Zhejiang, China; ^3^Zhou Enlai School of Government, Nankai University, Tianjin, China; ^4^School of Philosophy, Zhongnan University of Economics and Law, Wuhan, Hubei, China

**Keywords:** overage labor, intergenerational financial support, older rural residents, depression, China

## Abstract

**Background:**

Depression is a major factor affecting the happiness of older rural residents. With the increasing aging of the Chinese population, overage labor is becoming more prevalent in rural areas of China. This study aimed to assess whether, and if so, how, overage labor affects depression status in older rural residents.

**Methods:**

Using data from the China Health and Retirement Longitudinal Study, this study explored the association between overage labor and depression among older rural residents by using ordinary least squares and moderated mediation models.

**Results:**

The results show that overage labor significantly reduced levels of depression in older rural residents. This result remained robust after using propensity score matching and double machine learning. Furthermore, the improvement of older rural residents' depression via overage labor is mainly achieved through work income, but this mediating effect is negatively moderated by intergenerational financial support. This implies that in traditional Chinese rural society, intergenerational financial support from children plays an important role in reducing depression among older rural residents.

**Conclusion:**

Our findings have potential policy implications for China and other developing countries in terms of addressing issues related to aging and depression in older adults.

## 1. Introduction

Depression is a common mental disorder. It is characterized by persistent sadness and a lack of interest or pleasure in previously rewarding or enjoyable activities. It can also disturb sleep and appetite. Tiredness and poor concentration are common.^1^ Depression affects more than 350 million people and is recognized as a leading cause of disease worldwide (1). In terms of prevalence, depression is regarded as the second most common disease globally after coronary artery occlusion (2) and is regarded as a core risk factor for suicidal behavior (3, 4). Studies have shown that effective intervention and aggressive treatment of depression can reduce suicidal ideation and behavior by 40–50% (5). By occupation, the probability of depression is significantly higher in rural residents than in non-rural residents (6, 7), and the suicide rate is 3.5 times higher for rural residents and farm owners than for the general population (8). Furthermore, the risk of suicide is significantly higher in rural residents in comparison with non-rural residents (9). In terms of region, compared with urban older adults, rural older adults have more obvious depressive symptoms (10), and they are most prevalent among male rural residents over the age of 65 years (11). Therefore, what calls for attention is the depression status of rural older adults and the mechanisms that influence it.

In general, the academic community roughly divides the factors affecting depression in rural residents into two categories: positive and negative. Positive factors, including social support (12), financial support, a sense of belonging, and family caregiving (13), can help to suppress depressive ideas and behaviors among rural residents. Negative factors, including financial pressure (14), pesticide exposure and application (15), heavy workload, natural disasters, climate change, market fluctuations, conflicts within the family, and poor physical condition (13, 16, 17), can induce depressive thoughts and behaviors. For older rural residents, workability, job satisfaction, stress and health status, and emotional support from one's spouse are all important factors that influence depression (18). Among these factors, financial factors are very important, and along with pesticide exposure and application, climate change, and physical conditions, these are the four most-cited influences in academia (19). In one study, scholars have found the incidence of depression to be as high as 91.3% in poor older adults in rural areas (20). This implies that factors affecting rural residents' income—such as continued work after retirement—may play an important role in reducing depression levels among older rural residents (21). Continued work after retirement can be seen as an important manifestation of active aging. Active aging emphasizes the relationship between activity and health and is defined as improving the life quality of older adults by optimizing their opportunities for health, participation, and security.^2^

However, the impact of continued work on the mental health of retired older adults is controversial. The Mental Health Promotion hypothesis suggests that continued work helps to reduce depressive symptoms and promote mental health levels in older adults by increasing their sense of job security (22) and improving their financial income, interpersonal relationships, and social status (23–26). For example, scholars have found that continued work can curb the depressive tendencies of urban retired older adults, but mainly through the mediating mechanism of the realization of social values (27). However, the Mental Health Impairment (or “No Impact” Hypothesis) suggests that continued work brings longer work hours, physically harder work (6), and takes away from older adults' leisure time, thereby reducing their life satisfaction (28). It has also been argued that continued work beyond the traditional expected retirement age is not associated with mental health (29). There is still controversy in the academic community regarding the effects of overage labor on depression in retired older adults and its mechanisms. In particular, to the best of our knowledge, the effects of overage labor on rural retired older adults and the mechanisms mediating such effects have not yet been examined.

Within existing research in this field, there is an objective regional imbalance. Developed countries, such as the United States, the United Kingdom, and Australia, have a much richer research output on the subject, with most conducted studies coming from these countries. Therefore, some scholars call for future research to focus on the mental health of rural residents in developing countries (9). The largest developing country in the world is China. By the end of 2021, the number of Chinese rural residents will be ~500 million, accounting for ~14.7% of the world's total rural popu lation^3^; among Chinese rural residents, there are ~119 million older rural residents aged 60 years and above, accounting for 23.8%, and ~89 million older rural residents aged 65 years and above, accounting for 17.7%. Yet, in contrast to the considerable size of China's rural population and its significant aging status, there is very little research on depression among older Chinese rural residents. A previous review found 167 representative international articles on this topic; however, only five studies were authored by Chinese scholars (19).

Compared with Western countries, rural China has different social security systems and social cultures. First, China's social security system is characterized by an urban–rural dual division. Most Chinese rural residents or migrant workers are part of the “informal economy” and do not enjoy the same high level of pension and healthcare as urban workers. Consequently, overage labor is very common in rural China as labor income plays a crucial role in supporting these rural residents. Second, there is a unique notion of intergenerational support prevalent in rural China, such as Yang “Er(nv) Fang Lao”.^4^ Under the traditional customs of rural China, children are expected to bear the living expenses of older adults, and a significant portion of income for rural “retired” older adults stems from the financial support provided by their children. This also provides emotional support for older adults in rural areas. Especially in the rural societies of China, children's “financial support” for their parents is not merely a form of material giving but also a manifestation of filial responsibility. In China, filial piety is a widely shared moral concept, considered an important standard for measuring a child's character and family cohesion. China's unique social security system and rural social culture provide an interesting perspective for observing the relationship between overage labor, intergenerational financial support, and depression in older rural residents. This could potentially serve as a significant supplement to research on depression among rural residents.

Therefore, this study investigated the following two questions: (1) Does overage labor affect depression in older rural residents, and if so, how? (2) Does financial support from children influence the mediating mechanisms between overage labor and depression among older rural residents, and if so, how? Using data from a sample of 22,625 Chinese rural residents aged 60–80 years, this study used ordinary least squares (OLS) and mediating effect models to predict the effect of overage labor on rural residents' depression levels and further analyzed the moderating effect of intergenerational financial support from their children. Meanwhile, the propensity score matching (PSM) (30)-mediated effect model proposed by Rosenbaum et al. was used to solve the non-random problem of rural overage labor to obtain the most realistic analytical results for this study. The data used in this study came from the China Health and Aging Tracking Survey (CHARLS), which was jointly implemented and completed by the Center for Healthy Aging and Development Research at Peking University and the Chinese Center for Disease Control and Prevention.

## 2. Core concepts and research hypothesis

### 2.1. Core concepts

#### 2.1.1. Overage labor

Overage refers to exceeding the legal retirement age. According to the Provisional Measures on the Retirement and Retirement of Workers (Guo Fa [1978] No. 104) issued by the State Council of China, the retirement age is 60 years for men and 50 years for women.^5^ In 1999, the Ministry of Labor and Social Security (now the Ministry of Human Resources and Social Security) raised the retirement age to 55 years for women cadres, based on financial development and social reality.^6^ Therefore, overage labor refers to working for a certain length of time beyond the legal retirement age. Compared to urban workers, the marker of “retirement” for rural residents is not significant, but as China has established a unified basic pension insurance system for urban and rural residents, all insured rural residents can start receiving their pensions when they turn 60 years old. Then, it is considered as the marker of “retirement” for rural residents.^7^

In academia, in addition to the concept of active aging, there are directly two concepts related to overage labor: productive aging and re-employment. Productive aging is intended to emphasize the positive aspects of aging, specifically the paid or voluntary production of goods and the provision of services in which older adults are involved. However, the concept does not explicitly qualify age. Retirement is just one example, and there is a greater emphasis on voluntary, unpaid work for older adults after retirement. Re-employment is the re-entry of an unemployed person into an employment relationship which generally entails remuneration. Overage labor is related to both concepts, albeit with some key differences. First, in the context of this study, the subject of overage labor is rural residents. Second, the age of overage labor is limited to after the national legal retirement age. Third, overage labor includes agricultural labor, non-agricultural self-employed labor, and non-agricultural hired labor. In this study, overage labor refers to rural residents aged 60–80 who have performed more than 104 h of total agricultural labor or more than 52 h of total non-agricultural labor in the past year.^8^

#### 2.1.2. Intergenerational financial support

Intergenerational support in family relationships is usually defined as upward support from children to parents and downward support from parents to children, in emotional, spiritual, and material terms. Intergenerational financial support is an important component of intergenerational support systems. Both in developed Western and East Asian societies, adult children are the long-term health guarantors and social support providers for their aging parents and, in most cases, form the backbone of their parents' support system (31, 32). Particularly in East Asia, the old pattern of father-son (daughter) cohabitation and financial support for aging parents has persisted (33). In rural China, the family tradition of “Yang Er (nv) Fang Lao.” is very popular, and the financial support of children plays a relevant role in the income of older adults. In the past, most older adults relied almost entirely on their children for financial security (34, 35), and even today, children still play a substantial role in supporting their parents financially when they age. Intergenerational financial support, as defined in this study, refers to the financial support received by overage laborers from their children in the past year.

### 2.2. Research hypothesis

#### 2.2.1. Overage labor and depression among older rural residents

“Overage labor” can affect the psychological state of older adults through both value realization and financial needs. At the level of value realization, overage labor helps older adults to maintain their role after “retirement” (21) and promotes the affirmation of self-worth, leading to positive attitudes toward aging (36). In addition, overage labor helps older adults to remain socially active and, through the maintenance and reconstruction of social relationships, it helps to improve older adults' evaluation and cognition of the self-aging process (37). In terms of financial demand, overage labor can not only increase income to obtain living security but also alleviate the “feeling of pressure” caused by the financial dependence of older adults on their children. Furthermore, it positively affects the mental health of older adults. Therefore, overage labor is generally helpful in relieving the psychological anxiety and depression levels associated with rural overage labor.

However, older adults in rural China have greater financial needs than retired older adults in urban areas. There are three reasons for this. First, most Chinese rural residents or migrant workers are in the “informal economy,” outside the scope of labor law protection, and are regarded as temporary laborers (38). As a result, most rural residents and migrant workers cannot enjoy the benefits provided by their employers in the same way as urban workers. Second, although China has established a unified basic pension insurance system for both urban and rural areas, the system provides a basic, low level of pension protection. It is difficult to compare the level of old-age protection for rural older adults with the benefits provided to urban “retired” older adults. Third, owing to the lack of a sound social security and pension system, Chinese farmers are more inclined to accumulate than to consume to prepare for their future retirement. Thus, income constitutes a very important “security” for older adults in rural China. Therefore, we believe that income is an important mediating variable in the impact of overage labor on the depression levels of older rural residents. In summary, we propose the following two hypotheses:

Hypothesis 1a: Overage labor is negatively associated with depression levels among older rural residents, and overage labor reduces the depression level of rural residents.

Hypothesis 1b: Labor income plays a partially mediating role between overage labor and depression levels among older rural residents.

#### 2.2.2. Moderated mediating effect: intergenerational financial support and depression among older rural residents

Previous studies have shown that family role is an essential element influencing depression in rural residents and that financial and emotional support from family can help reduce depression among rural residents (13). Due to the limited level of state retirement protection in rural China, financial support from children is an important part of parents' income in their later years; therefore, it is also vital for their mental health (39, 40). Financial support from children may help relieve the “life pressure” and “old-age anxiety” of older rural residents. In particular, in China's rural society, children's “financial support” for their parents is not just a material gift but also a manifestation of “filial responsibility.” In China, filial responsibility is seen as an important measure of children's character and family cohesion, and it is a moral concept widely shared by the Chinese people. From the perspective of older adults in rural areas, more financial support from children for their parents means more filial responsibility for the children and closer and more harmonious family relationships. Thus, financial support from children also implies emotional support that increases psychological satisfaction (41) of older adults, reducing their depressive symptoms (42). However, we should also notice that rural older adults who have received financial support from their children are also likely to be less dependent on overage labor income for mental health maintenance as a result. In other words, for older rural residents, financial support from children reduces their motivation to earn and depend on income through continued work, with the result that the depressive effect of work income on older rural residents' depression is correspondingly reduced.

Hypothesis 2: The mediating role of work income on the relationship between overage labor and depression among older rural residents is negatively influenced by the moderating effect of intergenerational financial support from children.

## 3. Methods

### 3.1. Data

This study used data from the China Health and Retirement Longitudinal Study (CHARLS), which was jointly conducted and completed by the Center for Healthy Aging and Development Research at Peking University and the Chinese Center for Disease Control and Prevention. CHARLS data are widely used for research on issues pertaining to old age, health, and labor and are characterized by having a large sample, longitudinal dimension, and wide coverage. It also covers key relevant respondent information, such as household structure and employment, retirement, and health status. It includes a wide range of labor market participation indicators and a rich set of personal health status indicators, which are nationally representative and can fully reflect the personal health and labor participation status of China's rural older residents. In the specific data cleaning process, this study used the Harmonized CHARLS dataset collated by Peking University. It selected surveys from 2011, 2013, 2015, and 2018 to form a mixed cross-section of data. Per the research topic, the study was limited to older adults aged between 60 and 80 years with rural hukou (household registration)—a unique Chinese identity system that can be used to identify whether respondents are rural residents or not (43). After excluding samples with missing and rejected responses for key variables, the final valid sample size obtained for this study was 22,625. The empirical analysis of this study was completed using Stata (version 16) and SPSS (version 28).

### 3.2. Variable measurements

#### 3.2.1. Dependent variable

The dependent variable of the study, depression, was measured using a simplified version of the Center for Epidemiologic Studies Depression Scale (CES-D10). CES-D is designed to address the frequency of current depressive symptoms and focuses on measuring individual depressive effects with good reliability and validity. The CES-D10 is a simplified version of the CES-D and is completed by asking the participant to state the frequency of 10 depression-related symptoms in the past week. Answers include four frequencies: “Occasionally or none (<1 day)”; “Sometimes (1–2 days)”; “Often or half the time (3–4 days)”; and “Most of the time or constantly (5–7 days)”. Each item was marked from 0 to 3, and the final depression score obtained was between 0 and 30. The higher the depression score, the more severe the individual's depressive symptoms and poorer mental health.

#### 3.2.2. Independent variable

The independent variable of the study is the labor supply status of rural older adults. There are various definitions of older adults' participation in the labor force, such as the total days or hours worked by older adults in the past year. We referred to the definition by Chen et al. (44) and used the total non-agricultural labor time of 52 h in a year and the total agricultural labor time of 104 h in a year as the threshold; thus, we defined older adults with a total non-agricultural labor time exceeding 52 h or total agricultural labor time exceeding 104 h in the past year as overage labor and assigned a corresponding value of 1. Otherwise, they were regarded as retired older adults out of labor status, with an assigned value of 0 (44). In addition, we further classified overage labor into agricultural, non-agricultural self-employed, and non-agricultural employed labor based on relevant respondent responses.

#### 3.2.3. Control variables

Focusing on depression of older adults and referring to existing research (45), we selected individual and household-level control variables. The control variables at the individual level included the following: age, with age squared added to control for the non-linear effects of age; gender, with male assigned 1 and female assigned 0; marriage, with spouse assigned 1 and no spouse assigned 0; education, with below junior high school assigned 1, high school and technical school assigned 2, and higher education assigned 3; health, with hospitalization in the past year assigned 1 and no hospitalization assigned 0; pension, with pension assigned 1 and no pension assigned 0; and social work, with social work in the past year assigned 1 and no social work assigned 0. Household-level control variables included family size, number of people in the household, and child contact with a value of 1 assigned for contact with children and 0 for no contact with children and household dwelling assigned a value of 1 for living in a rural area and 0 for living in an urban community.

It is worth noting that due to the limitations of data availability, this study was not able to control for some variables that may affect the estimated results. For example, the attitudes of older adults toward aging may influence both depression and labor supply decisions among older adults, with those who held negative attitudes toward aging experiencing more depression and being more inclined to withdraw from the labor market. We left this question for further study.

#### 3.2.4. Mediating and moderating variables

Based on the previous analysis, this study chose work income as a mediating variable for labor to alleviate depressive symptoms in older adults. Income was measured using older adults' earned income from work and was conventionally log-transformed. In addition, the effect of work income on depressive symptoms in older adults may be moderated by children's financial support. The role of work income in reducing depression in older adults may be somewhat diluted in the presence of financial support from children. Therefore, in this study, financial support from children was chosen as the moderating variable in the moderated mediation model. Individuals who received financial support from children in the previous year were assigned a value of 1; otherwise, they were assigned a value of 0.

### 3.3. Model selection

#### 3.3.1. Ordinary least square model

Considering that the dependent variable of the study—depression—is a continuous, normally distributed variable and that several confounding factors were controlled for in the study, we first used an OLS model to estimate the effect of overage labor on depression in rural older adults. The model was set as follows.


(1) $$\begin{array}{l} depress_{i}=\alpha_{0}+{\;\beta }_{0}work_{i}+\sum \gamma_{m}X_{mi}+\varepsilon_{i}\;\# \end{array}$$

where *depress*_*i*_ denotes the depression status of the i-th rural older adult, *work*_*i*_ denotes whether the i-th rural older adult is overage labor, *X*_*mi*_ denotes other control variables, ε_*i*_ denotes the random error term, and β_0_ denotes the effect of overage on depression in rural older adults.

#### 3.3.2. Propensity score matching

The labor supply behavior of rural older adults was not random; however, it could be influenced by several factors. This means that there might be self-selection effects among overage labor and that differences in depression between overage labor and retired older adults out of the labor force might arise from factors that affect older adults' labor supply rather than labor supply itself. Therefore, it is difficult to obtain unbiased estimation results if only OLS regression is used. To solve the non-random problem of rural overage labor, we used PSM (30). The basic principle of this method is to match individuals in the treatment group with individuals in the control group who are as similar as possible based on the probability of obtaining treatment, thus achieving a randomized treatment. In this study, the treatment group was overage labor, and the control group was retired older adults. The specific steps are as follows.

In the first step, a logit model was used to screen for variables that affect the labor supply of rural older adults.

In the second step, the propensity score for overage labor among older adults was calculated by the logit model based on the screened variables.


(2) $$\begin{array}{l} \begin{array}{l} PS(\text{X})=P_{r}\{D=1|\text{X}\}=E\{D|\text{X}\}\# \end{array} \end{array}$$

where D represents whether the older adult is overage labor, in which case D = 1, otherwise D = 0; and **X** represents the covariates that affect an individual becoming overage labor. Given **X**, the probability of older adults being overage labor is equal.

In the third step, nearest neighbor matching, kernel matching, and radius matching were used to match the treatment and control groups based on propensity scores and to calculate the depression effect of overage labor.

#### 3.3.3. Double machine learning

This study aimed to investigate the impact of overage labor on depression among older adults. However, overage labor was closely associated with covariates such as individual health status and family financial conditions. Without controlling for these covariates, it is difficult to accurately identify the actual effects of overage labor on depression in older adults. Unfortunately, traditional econometric models and methods may not be effective in handling high-dimensional data with a limited sample size. The main reasons include the curse of dimensionality, multicollinearity, and limited control over crucial covariates, resulting in biased estimates.

In contrast, machine learning models provide effective estimation in high-dimensional data scenarios, thus avoiding the curse of dimensionality. However, machine learning models often employ regularization techniques to achieve dimensionality reduction. Although these techniques help reduce variance, they also introduce estimation bias, known as regularization bias. To address this issue, Chernozhukov et al. (46) proposed the Double Machine Learning (DML) method, which utilized machine learning models multiple times in auxiliary equations to eliminate regularization biases. This method has several advantages. First, it overcomes the curse of dimensionality associated with controlling numerous variables in traditional linear regression. Second, the DML method does not require pre-specification of the relationships between variables, as non-parametric machine learning models can handle non-linear relationships, thus improving estimation accuracy. Building upon these advantages, this study further adopted a double machine learning model to assess the effects of overage labor on depression. The specific model setup was as follows:


(3) $$\begin{array}{l} depress_{i}=\delta_{0}work_{i}+g\left(\text{X}\right)+U,E\left[U|work,X\right]=0\;\# \end{array}$$

(4) $$\begin{array}{l} work_{i}=h\left(\text{X}\right)+V,E\left[V|\text{X}\right]=0,\;\# \end{array}$$

where *depress*_*i*_ represents the depression status of individual i, *work*_*i*_ indicates their participation in overage labor, and X represents a series of control variables. The specific forms of g (X) and h (X) are unknown, but their estimations can be computed using machine learning method. U and V are disturbance terms, and δ_0_ represents the treatment effect of interest.

#### 3.3.4. Moderated mediation model

Referring to Preacher et al. (47), we constructed the following moderated mediation model.


(5) $$\begin{array}{l} M_{i}=\alpha_{1}+\beta_{1}work_{i}+\sum \gamma_{m}X_{mi}+\varepsilon_{i}\;\# \end{array}$$

(6) $$\begin{array}{l} \begin{array}{c} depress_{i}=\alpha_{2}+\beta_{2}work_{i}+\theta_{0}M_{i}+\theta_{1}W_{i}+\theta_{2}M_{i}W_{i}\\ +\sum \gamma_{m}X_{mi}+\varepsilon_{i}\;\# \end{array} \end{array}$$

where *M*_*i*_ and *W*_*i*_ are the older adults' work income and children's financial support, respectively. The value of the conditional indirect effect is α_1_(θ_0_+θ_2_*W*_*i*_), which indicates the mediating effect of work income on depression, moderated via children's financial support.

## 4. Results

### 4.1. Descriptive statistics

Table 1 shows the results of descriptive statistics. The average depression score of Chinese rural older adults was 9.135, which is close to the critical value of 10, indicating that the depression status of Chinese rural older adults requires attention. Regarding labor supply among older adults, 54.2% of rural older adults in China were overage labor. This shows that overage labor is quite common in rural areas. Regarding other characteristics of the sample, the average age was 67 years; the gender ratio was balanced, with 48.5% of men; the vast majority of older adults had a spouse, accounting for 81.3%; the percentage of those who have been hospitalized in the past year was 15.7%; approximately 70% of older adults received a pension; only 41.7% of older adults were involved in social activities; 87.7% older adults maintained contact with their children; 79.8% older adults received their children's financial support. In terms of differences between overage labor and retired older adults, overage labor had significantly lower levels of depression, while there were significant differences between the two in areas including age, gender, and educational attainment. These findings provide a basis for further research.

**Table 1:** Descriptive statistics.

**Variables**	***n***	**Full sample**		**Labor status**		**Differences**
		**Mean**	**Standard deviation**	**Overage labor**	**Retired older adults**	
**Dependent variable**					
Depress	22,625	9.135	6.531	8.734	9.611	−0.877^***^
**Independent variable**					
Work	22,625	0.542	0.498			
**Control variables**					
Age	22,625	66.981	5.363	65.758	68.431	−2.673^***^
Age^2^	22,625	45.153	7.365	43.463	47.155	−3.692^***^
Gender	22625	0.485	0.500	0.553	0.404	0.148^***^
Marriage	22,625	0.813	0.390	0.870	0.747	0.123^***^
Education	22,625	1.031	0.185	1.033	1.028	0.005^**^
Health	22,625	0.157	0.364	0.125	0.194	−0.069^***^
Pension	22,625	0.712	0.453	0.715	0.708	0.007
Social work	22,625	0.419	0.493	0.401	0.440	−0.039^***^
Family size	22,625	3.086	1.709	3.137	3.025	0.112^***^
Children connect	22,625	0.877	0.328	0.866	0.891	−0.025^***^
Household dwelling	22,625	0.754	0.431	0.797	0.704	0.093^***^
**Mediating and moderating variables**					
Work income	22,625	1.091	2.898	1.647	0.432	1.215^***^
Intergenerational financial support	22,625	0.798	0.402	0.793	0.803	−0.009^*^

^*^, ^**^, and, ^***^ indicate significance at the 10%, 5%, and 1% levels, respectively.

### 4.2. Benchmark regression

Table 2 shows the results of the benchmark regression using the OLS model. Model 1—a regression of depression directly on labor status—showed that overage labor had lower levels of depression, significant at the 1% level. Model 2 controlled for a range of individual and household-level variables based on Model 1. The regression results showed that, when holding other variables constant, the depression score of overage labor was significantly lower (0.503). This suggests that among rural Chinese older adults, overage labor had significantly lower levels of depression than retired older adults. Thus, Hypothesis 1a was supported.

**Table 2:** Benchmark regression results.

	**Model 1**	**Model 2**	**Model 3**	**Model 4**	**Model 5**
	**Depress**	**Depress**	**Depress**	**Depress**	**Depress**
Work	−0.866^***^	−0.503^***^			
	(0.087)	(0.090)			
Agricultural labor			−0.263^***^		
			(0.097)		
Non-agricultural self-employed labor				−1.489^***^	
				(0.215)	
Non-agricultural employed labor					−2.230^***^
					(0.166)
Age		0.719^***^	0.577^***^	0.614^**^	0.456
		(0.200)	(0.215)	(0.299)	(0.282)
Age^2^		−0.544^***^	−0.451^***^	−0.476^**^	−0.365^*^
		(0.146)	(0.156)	(0.215)	(0.204)
Gender		−2.000^***^	−1.894^***^	−1.556^***^	−1.595^***^
		(0.086)	(0.094)	(0.137)	(0.132)
Marriage		−1.352^***^	−1.363^***^	−1.223^***^	−1.244^***^
		(0.121)	(0.128)	(0.169)	(0.163)
Education		−0.965^***^	−1.177^***^	−0.665^**^	−0.746^***^
		(0.204)	(0.243)	(0.333)	(0.282)
Health		2.251^***^	2.232^***^	2.020^***^	1.946^***^
		(0.122)	(0.130)	(0.174)	(0.173)
Pension		−0.389^***^	−0.303^***^	−0.667^***^	−0.596^***^
		(0.104)	(0.114)	(0.162)	(0.154)
Social work		−0.927^***^	−0.984^***^	−1.415^***^	−1.265^***^
		(0.084)	(0.092)	(0.133)	(0.125)
Family size		−0.038	−0.056^**^	−0.092^**^	−0.105^***^
		(0.025)	(0.027)	(0.040)	(0.038)
Children connect		−0.875^***^	−0.792^***^	−0.795^***^	−0.694^***^
		(0.135)	(0.146)	(0.233)	(0.212)
Household dwelling		1.313^***^	1.264^***^	1.610^***^	1.431^***^
		(0.096)	(0.108)	(0.142)	(0.132)
_cons	10.180^***^	−10.413	−4.883	−6.718	−1.077
	(0.107)	(6.835)	(7.380)	(10.328)	(9.731)
*N*	22,625	22,625	19,416	9,643	10,532
*r* ^2^	0.007	0.073	0.066	0.076	0.091

Robust standard errors are in parentheses; ^*^, ^**^, and ^***^ indicate significance at the 10%, 5%, and 1% levels, respectively. Depress, depression.

The depressive status of rural older adults was also influenced by individual- and household-level factors. Regarding individual characteristics, the relationship between depression and age showed an inverted U-shaped curve, with depression levels in older adults rising and then falling with age between the ages of 60 and 80 years. Depression levels were lower among older adults who were male, had a spouse, and had higher levels of education. Hospitalization status in the past year significantly increased depression scores in older adults. Receiving a pension and participating in social activities significantly reduced depression scores in older adults. At the household level, family size—although negatively associated with depressive status among older adults—did not significantly affect depression; maintaining contact with their children significantly reduced depression in older adults; and those who remained in rural areas had significantly lower levels of depression compared to those living in urban communities.

Considering the possible differences in the effects of different types of labor to alleviate depression in older adults, we further divided overage labor into agricultural, non-agricultural self-employed, and non-agricultural employed labor to explore the differences in depression between them and retired older adults. Models 3–5 (Table 1) reported the differences in depression status of agricultural labor, non-agricultural self-employed labor, and non-agricultural employed labor compared with retired older adults, respectively. It was found that overage labor had significantly lower levels of depression, regardless of the type of work. Specifically, compared to retired older adults, agricultural labor, non-agricultural self-employed labor, and non-agricultural employed labor had lower depression scores of 0.263, 1.489, and 2.230, respectively.

### 4.3. Double machine learning

Given the endogeneity issues potentially inherent in OLS estimation, this investigation adopted a DML methodology to alleviate such complications. Precisely, the randomly selected sample was partitioned into quintiles, with the kth segment (where k ≤ 5) functioning as the auxiliary sub-sample, while the remainder comprised the primary sub-sample. In the auxiliary sub-sample, we employed random forest (RF) and support vector machine (SVM) techniques for the purpose of estimating the influence of the control variables on both the outcome and the predictor variables. This allowed for the derivation of the residuals from the predictor variables, as well as the residuals from the outcome variables. Subsequently, within the primary sub-sample, we applied a regression model to the residuals of the outcome variable, using the residuals of the predictor variable, with the aim of estimating the treatment effect. This sequence was repeated for each of the five segments, and the calculated treatment effects were subsequently averaged to yield an overall average treatment effect as per the DML estimation.

Table 3 elucidates the estimation outcomes obtained through the application of diverse machine learning methodologies. The findings represented in Table 3 consistently indicate that, regardless of the machine learning method implemented, overage labor exhibits significantly reduced instances of depression.

**Table 3:** Double machine learning estimation results.

**Estimation method for the dependent variable**	**RF**		**SVM**	
Estimation method for the independent variable	RF	SVM	RF	SVM
	−0.442^***^	−0.446^***^	−0.419^***^	−0.542^***^
St.Err.	0.090	0.093	0.086	0.088
*N*	22,625	22,625	22,625	22,625

^*^, ^**^, and ^***^ represent statistical significance at the 10%, 5%, and 1% levels, respectively.

### 4.4. Robustness tests

**Figure 1:**
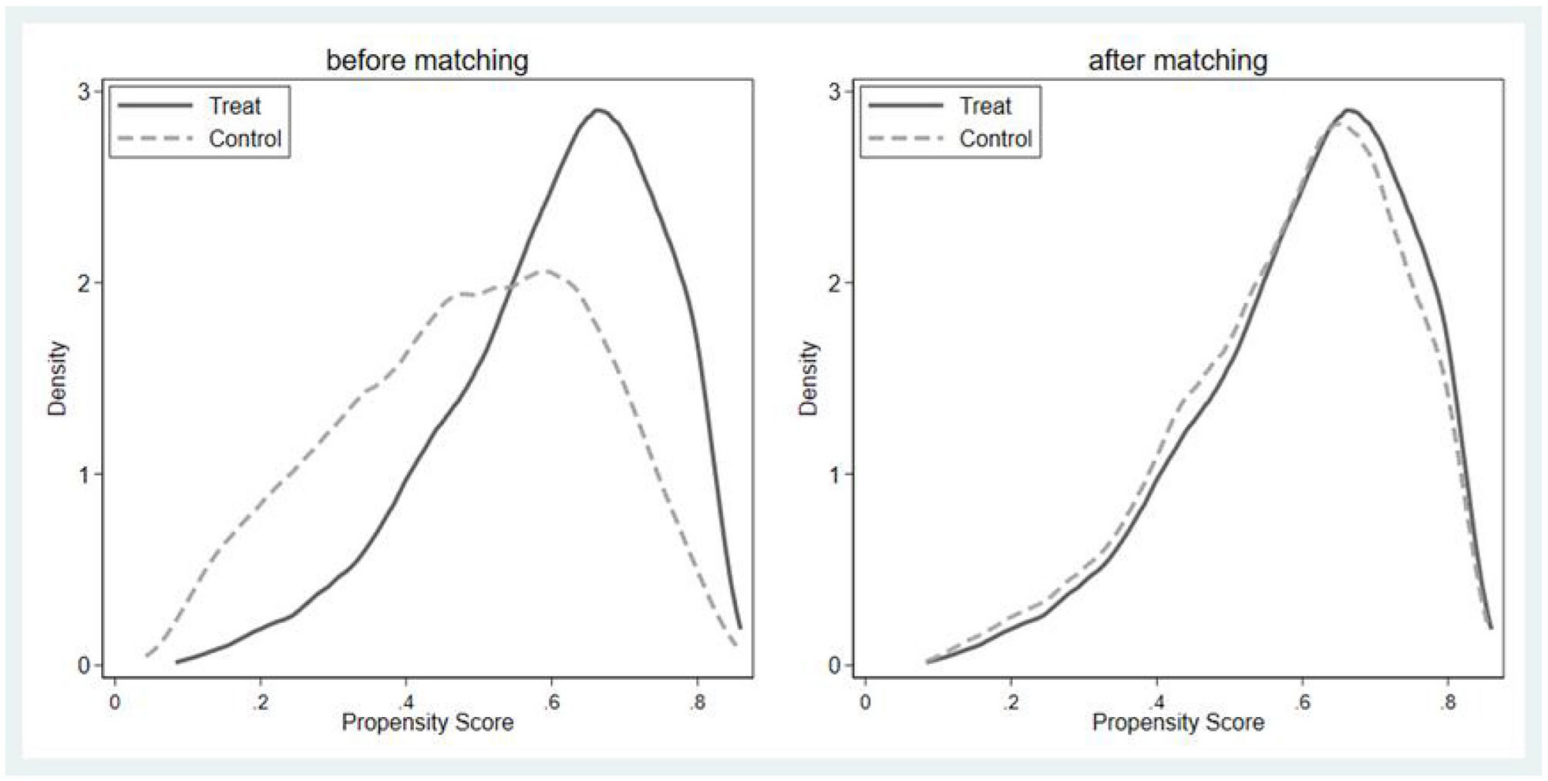
Balance test.

The above results suggest that overage labor elicits lower levels of depression than retired older adults. However, this finding might be unreliable owing to the non-random nature of the labor supply of older adults. Therefore, we further used PSM to address the self-selection problem. First, we added intergenerational financial support, satisfaction, and exercise to the original control variables and then used a logit model to filter variables that influence overage labor. Based on the logit estimation results, we screened out two insignificant variables, namely family size and children's financial support, and included the remaining variables in the covariates affecting the labor supply of older adults. Second, the reliability of the matching results was tested. The effective implementation of PSM presupposes a balance test, which is a similar distribution in propensity scores between the treatment and control groups after matching. Figure 1 demonstrates that before the implementation of matching, there is a large difference in the distribution of propensity scores between the treatment group and the control group, while after the implementation of matching, the curves of the two largely overlap. This proves that the balance test is passed. Finally, the net effect of labor supply on older adult depression is estimated using multiple matching methods. The estimation results are more reliable (Table 4).

**Table 4:** Results of covariate screening.

**Variables**	**Coefficients**	**Standard errors**	***t*-value**	***p*-value**	**Significances**
Age	0.265	0.072	3.68	0	^***^
Age^2^	−0.263	0.053	−5.01	0	^***^
Gender	0.607	0.030	20.53	0	^***^
Marriage	0.434	0.039	11.00	0	^***^
Education	−0.175	0.079	−2.22	0.027	^**^
Health	−0.475	0.040	−11.96	0	^***^
Pension	0.081	0.033	2.44	0.015	^**^
Social work	−0.165	0.029	−5.63	0	^***^
Family size	−0.008	0.009	−0.89	0.373	
Children connect	−0.180	0.046	−3.95	0	^***^
Household dwelling	0.567	0.034	16.86	0	^***^
Intergenerational financial support	−0.020	0.037	−0.54	0.593	
Satisfaction	0.051	0.018	2.76	0.006	^***^
Exercise	0.331	0.029	11.30	0	^***^

Robust standard errors are in parentheses; ^**^, and ^***^ indicate significance at the 5% and 1% levels, respectively.

The average treatment effects under different matching methods are reported in Table 5. Among them, the k-nearest neighbor matching results indicate that overage labor had a 0.259 lower depression score compared to retired older adults; the kernel matching results are similar to the radius matching results, indicating that overage labor has a 0.5 lower depression score. These estimates are consistent with the original findings.

**Table 5:** Average treatment effects.

**Matching methods**	**Treatment group**	**Control group**	**Net effect**	**Bootstrap standard error**	***t*-value**
k-nearest neighbor matching	8.710	8.970	−0.259^**^	0.119	−2.17
Kernel matching	8.710	3.968	−0.509^***^	0.124	−5.06
Radius matching	8.710	3.936	−0.504^***^	0.127	−4.94

The standard error in column 5 is obtained by bootstrapping 500 times. ^**^ and ^***^ indicate significance level at 5% and 1%, respectively.

In addition, considering that a high proportion of rural overage labor will affect the accuracy of the estimation results when defining overage labor by total agricultural labor time over 104 h or total non-agricultural labor time over 52 h, we adopted a stricter definition of labor time to conduct robustness tests. Table 6 presents the results of robustness tests under different definitions of labor. Model 6 showed the estimation results for increasing the labor time threshold by five times, while model 7 showed the estimation results for increasing it by ten times. With the adoption of a stricter definition of labor hours, rural overage labor still had significantly lower levels of depression than retired older adults. These robustness tests all remain consistent with the original findings, providing ample evidence that rural overage labor leads to lower levels of depression than retired older adults.

**Table 6:** Replacement labor definition test.

	**Model 6**	**Model 7**
	**Depress**	**Depress**
Work	−0.435^***^	−0.345^***^
	(0.089)	(0.093)
Control variables	yes	yes
*N*	22625	22625
*r*2	0.072	0.072

^***^ indicates significance at the 1% level. Depress, depression.

### 4.5. Moderated mediation effect

Considering the weak validity of the stepwise method in testing mediating effects, we choose the Bootstrap method, which has been considered more reliable in recent years, to test for mediating effects (48). The results are shown in Table 7. The interval between the upper and lower values of the Boot confidence interval (CI) did not include 0 for either the total, direct, or indirect effects, indicating that work income played a partially mediating role between labor supply and the depression status of older adults. Hypothesis 1b was verified.

**Table 7:** Mediation effect test.

**Mediation variable**	**Effect**	**Effect value**	**Bootstrap standard error**	**LLCI**	**ULCI**
Work income	Total effect	−0.500	0.090	−0.674	−0.325
	Direct effect	−0.352	0.090	−0.528	−0.175
	Indirect effect	−0.148	0.014	−0.176	−0.120

Upper and lower boot confidence interval data were measured at *p* < 0.

To test whether there is a moderated mediating effect of children's financial support, we used the Bootstrap method to test the moderated mediating effect model, which describes the mediating effect of work income separately under the condition of having or not having children's financial support. From the results in Table 8, the indirect effect of labor supply on depression through work income was−0.204 (CI = [−0.259, −0.150]) when older adults received intergenerational financial support. The indirect effect of labor supply through work income on depression was −0.133 (CI = [−0.162, −0.103]) when older adults enjoyed intergenerational financial support, with CIs not including 0. The indirect effect was greater when there was a lack of intergenerational financial support. The indirect relationship between older adults' labor supply through work income and depression was 0.072 (CI = [0.014, 0.013]), and the CI did not include 0. This suggests that the mediating role of work income in the relationship between labor supply and depression was negatively influenced by the moderating effect of children's financial support. Thus, Hypothesis 2 was supported.

**Table 8:** Tests for moderated mediation effect.

**Mediation variable**	**Conditional indirect effects**					**Moderated mediation index**			
	**Condition**	**Effect value**	**Bootstrap standard error**	**LLCI**	**ULCI**	**Index**	**Bootstrap standard error**	**LLCI**	**ULCI**
Work income	Intergenerational financial support = 0	−0.204	0.028	−0.259	−0.150	0.072	0.030	0.014	0.013
	Intergenerational financial support = 1	−0.133	0.015	−0.162	−0.103				

LLCI, lower-level confidence interval; ULCI, upper-level confidence interval.

## 5. Discussion

The impact of overage labor on depression in older adults remains controversial (6, 22, 24, 28). This study validated the controversy using data from rural China. Overall, the findings indicated that overage labor reduces depression levels among older adults in rural areas.

### 5.1. Overage labor reduces depression levels among older adults by increasing their financial income

Increasing financial income helps reduce depression levels among older adults (13, 15). Particularly in China, although a unified basic pension insurance system has been established for both urban and rural areas, there is a significant disparity between them. The basic pension insurance system, as a safety net, fails to substantially improve the financial conditions of older adults in rural areas. The amount of national pension received by older rural adults is far from sufficient to sustain their livelihoods. According to data from the National Bureau of Statistics of China, in 2019, the average pension for urban residents was approximately 2,300 RMB per month, while for rural residents, it was approximately 150 RMB per month, making the former approximately 15 times higher than the latter. As of 2021, the average annual pension for rural older adults in China was 2,291 RMB, while the average annual consumption expenditure per rural resident was 15,916 RMB (49). By the end of 2021, there were still 34.74 million rural older adults receiving the minimum living allowance and 4.38 million rural older adults receiving assistance for destitute individuals.^9^ Due to insufficient social security coverage, it is common to see a large number of older adults in rural China engaging in various forms of labor. Their aim is to enhance their financial situation, alleviate the burden on their children, and ease the pressure of aging by continuing to work. This study empirically examined data from rural China and confirmed that overage labor, by increasing the financial income of older adults in rural areas, reduces their levels of psychological depression.

### 5.2. Intergenerational financial support has a negative moderating effect on the mediating role of financial income

However, rural China has a unique social culture. Confucian ethics, with filial responsibility at its core, still dominate Chinese family relations and form an important basis for social norms and evaluation. In Weber's view, filial responsibility is the source of all virtue in Chinese civilization, and it is not the ideals of an order of the commonwealth or the happiness that portray the character of Chinese civilization (50), but the ethics of identity embodied by filial responsibility (51). An important manifestation of the filial responsibility ethic in rural China is the concept of “Yang Er (nv) Fang Lao.” This concept assumes that parents are responsible for raising their children to adulthood. As parents move into their later years, it is the children's obligation to provide them with the appropriate financial support. This concept encompasses both “authoritative” and “emotional” filial logic. The former is based on the status authority and hierarchical order of the parent in relation to the offspring; the latter is based on the natural kinship between the parent and the offspring (52). With the interplay of both authoritative and emotional filial logics, “Yang Er (nv) Fang Lao” not only has a strong basis of legitimacy but also becomes a binding shared concept that regulates the behavior of rural society members. Especially with the rapid advancement of urbanization in China, a large number of young people have begun to migrate away from rural areas, leaving behind a significant population of older adults. According to statistics, in 2020, there were approximately 16 million left-behind older adults in rural China.^10^ The influx of rural young people into cities has increased the distance between the younger and older generations in terms of caregiving and support. The separation between generations has resulted in the financial support provided by the younger generation replacing daily companionship and care, becoming an important way to demonstrate filial piety. As a result, the financial support from the younger generation carries multiple meanings. From the perspective of the older generation, the financial support from their children represents not only material assistance but also a display of care, filial piety, and emotional support. It is also seen as a measure of the success of their family education and relationships (52). Intergenerational support has become an important aspect of social capital within family relationships (53). The financial support provided by children helps weaken the dependence of older adults in rural areas on continued labor for income generation.

Although this study provided several important insights, its limitations should be noted. First, owing to data availability limitations, this study was unable to control for some variables that may affect the estimated results. For example, older adults' attitudes toward aging may affect both their depressive status and labor supply decisions, whereby those who have negative attitudes toward aging may experience depressive feelings more frequently and may be more likely to exit the labor market. This issue will be addressed in a subsequent study. Second, this study did not examine the mediating effects of overage labor on older rural residents' depression. As shown in Table 5, overage labor still had a significant effect on older rural residents' depression status after controlling for work income, suggesting that other mediating mechanisms also exist between overage labor and depression. Third, the types of work for which older rural residents perform overage labor may be further refined. For example, the effects of participation in agricultural vs. non-agricultural labor on older rural residents' depression may differ. In addition, the important factor of working hours was not examined in this study. In future, more refined studies based on the type of work and related characteristics will better elucidate how overage labor affects depression levels.

## 6. Conclusion and policy implications

China is facing a serious aging challenge, particularly evident in rural areas. With the influx of young people into cities, the age structure of China's rural population is characterized by the “hollowing out” of older adults and children. These older rural residents often choose to continue working in agriculture or non-agriculture after reaching their legal retirement age. This poses the question of how this may affect depression. There is no consensus in the literature regarding the mental health effects of overage labor. Therefore, this study provided empirical evidence from China by examining the relationship between overage labor and depression among older rural residents. The empirical results suggest that overage labor significantly improves depression among older rural residents, with work income indirectly influencing this relationship. This indirect effect is negatively moderated by financial support from children. These findings have important policy implications for China and other developing countries dealing with the issues of aging and depression in older adults and can be utilized to address these issues.

The policy implications of the above findings can be designed to address both financial and socio-cultural aspects. In terms of the economy, the following policy measures can be considered. First, establishing an inclusive and equitable social security system is of paramount importance. Enhancing the social security level for older rural residents can help alleviate the psychological pressure and anxiety caused by financial burdens. Second, improving the working conditions of older rural residents and safeguarding their labor rights can unlock the labor potential of this group and promote their active aging in rural areas. From a socio-cultural perspective, the following policy recommendations can be made. First, it is important to guide public opinion to view the phenomenon of overage labor among older rural residents in a correct manner, eliminating biases and misconceptions. This will enable them to be recognized as important contributors to productive activities. Second, promoting and preserving the traditional culture of respecting and caring for older adults can be achieved through building a social support system based on the family unit, strengthening emotional support from younger generations to their parents. Lastly, attention should also be given to exploring alternative sources of emotional support from sources other than their children. This can be achieved by establishing “organizational emotional support” that complements the emotional support from their children through policy provisions and public service initiatives, for example, enhancing community-based familial cultural activities, improving home-based social work services, and engaging volunteers in supporting the older rural residents.

## Data availability statement

The original contributions presented in the study are included in the article/supplementary material, further inquiries can be directed to the corresponding author.

## Author contributions

LH and YC: conceptualization and formal analysis. KW: methodology, visualization, and software. KW, JZ, and ZZ: validation. YC: investigation and project administration. ZZ: resources. JZ: data curation. KW, JZ, ZZ, and JW: writing—original draft preparation. LH, KW, JZ, ZZ, and JW: writing—reviewing and editing. LH: supervision and funding acquisition. All authors have read and agreed to the published version of the manuscript.

## Footnotes

^1^World Health Organization, https://www.who.int/health-topics/depression#tab=tab_1.

^2^World Health Organization, Active Aging: A Policy Framework, the WHO website: https://apps.who.int/iris/handle/10665/67215, April, 2002, p. 12.

^3^Data sources: China National Economic and Social Development Statistical Bulletin 2021, National Bureau of Statistics of China. Retrieved from http://www.stats.gov.cn/tjsj/zxfb/202202/t20220227_1827960.html. World Bank. https://data.worldbank.org.cn/indicator/SP.RUR.TOTL.

^4^“Yang Er(nv) Fang Lao” means raising children for retirement safeguard.

^5^Resolution of the Standing Committee of the National People's Congress on the Approval of the Provisional Measures of the State Council on the Retirement and Retirement of Workers, BYU Law Database, https://www.pkulaw.com/law?isFromV5=1.

^6^Notice on Stopping and Correcting Issues Relating to Early Retirement of Enterprise Employees in Violation of State Regulations, Ministry of Human Resources and Social Security of the People's Republic of China, http://www.mohrss.gov.cn/xxgk2020/fdzdgknr/zcfg/gfxwj/shbx/201407/t20140717_136210.html.

^7^Opinions of the State Council on the Establishment of a Unified Basic Pension Insurance System for Urban and Rural Residents, State Development [2014] No. 8, Central People's Government of the People's Republic of China, http://www.gov.cn/zhengce/content/2014-02/26/content_8656.htm.

^8^In fact, we have used a different definition of overage labor for robustness tests, increasing the working time threshold by factors of 5 and 10, respectively, with the results remaining unchanged. See the robustness tests section for more details.

^9^China statistical bulletin of the national economy and social development in 2021, http://www.stats.gov.cn/tjsj/zxfb/202202/t20220227_1827960.html.

^10^There are 16 million elderly people left behind in rural areas who will solve their pension problems? The first finance and economics, https://baijiahao.baidu.com/s?id=1714676922894143963&wfr=spider&for=pc.
